# Using Syndromic Surveillance to Investigate Tattoo-Related Skin Infections in New York City

**DOI:** 10.1371/journal.pone.0130468

**Published:** 2015-06-15

**Authors:** Mollie Kotzen, Jessica Sell, Robert W. Mathes, Catherine Dentinger, Lillian Lee, Corinne Schiff, Don Weiss

**Affiliations:** 1 New York City Department of Health and Mental Hygiene, Queens, New York, United States of America; 2 Centers for Disease Control and Prevention, Atlanta, Georgia, United States of America; 3 New York University, New York, New York, United States of America; Columbia University, UNITED STATES

## Abstract

In response to two isolated cases of *Mycobacterium chelonae* infections in tattoo recipients where tap water was used to dilute ink, the New York City (NYC) Department of Health and Mental Hygiene conducted an investigation using Emergency Department (ED) syndromic surveillance to assess whether an outbreak was occuring. ED visits with chief complaints containing the key word “tattoo” from November 1, 2012 to March 18, 2013 were selected for study. NYC laboratories were also contacted and asked to report skin or soft tissue cultures in tattoo recipients that were positive for non-tuberculosis mycobacterial infection (NTM). Thirty-one TREDV were identified and 14 (45%) were interviewed to determine if a NTM was the cause for the visit. One ED visit met the case definition and was referred to a dermatologist. This individual was negative for NTM. No tattoo-associated NTM cases were reported by NYC laboratories. ED syndromic surveillance was utilized to investigate a non-reportable condition for which no other data source existed. The results were reassuring that an outbreak of NTM in tattoo recipients was not occurring. In response to concerns about potential NTM infections, the department sent a letter to all licensed tattoo artists advising them not to dilute tattoo ink with tap water.

## Introduction

Tattoos have become popular in the United States (U.S.). A 2004 telephone survey found that 24% of those ages 18 to 50 years reported having a tattoo, including 36% of those ages 18 to 29 [1]. A 2012 Harris Interactive online poll indicated that 14% of U.S. adults had one or more tattoos in 2008, and this proportion increased to 21% in 2012 [2]. These reports suggest a considerable increase in tattoo prevalence in the U.S. in recent years.

The New York City (NYC) Department of Health and Mental Hygiene (DOHMH) licenses tattoo artists but not tattoo parlors. As of December 31^st^, 2012, there were 2,275 licensed tattoo artists in NYC. Artists must attend a course and pass an examination on infection control practices in order to obtain a license. There is no program to perform routine inspections of tattoo parlors. Artists are prohibited from tattooing anyone under 18 years of age.

Tattoo-associated complications include aseptic inflammatory reactions, acute regional lymphadenopathy, hematoma, and infections with organisms such as atypical mycobacteria, herpes compucturum, and hepatitis B and C [3,4]. In 2012, an outbreak of *Mycobacterium chelonae* infections affected 19 individuals tattooed by the same artist in Rochester, NY. The source was found to be premixed tattoo ink contaminated before distribution [5].

In May 2012 a community dermatologist reported a case of *M*. *chelonae* in a 22-year-old male tattoo recipient. The patient suspected that the artist had diluted the ink with tap water but was unwilling to divulge either the location or name of the tattoo artist. He alledged that a friend who resided upstate and had received a tattoo from the same artist and had a similar infection. Efforts to locate this second individual were not successful. When a second case of *M*. *chelonae* was reported to DOHMH in March 2013 in a tattoo recipient, we used emergency department (ED) syndromic surveillance data to search for additional tattoo-associated *M*. *chelonae* or other non-tuberculosis mycobacteria (NTM) infections. We also used syndromic surveillance data to describe the burden of tattoo-related ED visits (TREDV) in NYC from 2008 to 2012.

## Methods

### Ethics Statement

This study was part of an ongoing outbreak investigation in NYC and considered routine public health surveillance. Thus we did not submit this project to the NYC DOHMH Institutional Review Board.

### Case finding

DOHMH has operated an ED chief complaint syndromic surveillance system since 2001. Data from this system represent approximately four million ED visits annually (an estimated 98% of annual NYC ED visits) and include hospital name, date and time of visit, age, sex, chief complaint, international disease classification code (ICD-9 or 10) and disposition. We reviewed data transmitted daily from 49 of the 52 acute care hospitals in NYC during November 1, 2012 through March 31, 2013. Syndromic Surveillance case finding was limited to this time period to focus efforts on detecting a current outbreak. Visits with chief complaint containing the key word “tattoo,” including misspellings, were selected for study.

To identify possible cases of tattoo-related NTM infections, we obtained contact information of 31 TREDV cases from hospital medical records. A standardized questionnaire was administered to these cases to collect information on symptoms, duration, tattoo parlor, and artist. Completed questionnaires were reviewed by a physician for indications of NTM infection. TREDV patients who described an erythematous papular rash lasting two weeks or longer without significant improvement met the definition of possible NTM infection and were interviewed by a physician who then referred to a dermatologist for further evaluation.

As an adjunct to syndromic surveillance, NYC laboratories (hospital and large, commercial laboratories) were contacted and requested to report skin or soft tissue cultures positive for NTM from January 2012 through March 2013. Hospital laboratory directors reviewed skin and soft tissue NTM culture reports for indications that the infections were tattoo-related. Additionally, the NYC Health Code specifies that any unusual manifestation of disease in an individual, or a suspected outbreak involving 3 or more cases of a disease or condition not otherwise listed, should be reported to the health department. The investigation was undertaken in part to identify clusters of NTM in tattoo recipients, that traditional provider reporting might miss due to no single provider detecting more than two cases.

### Five-year TREDV trends

We exported and analyzed ED syndromic data from January 1, 2008 through December 31, 2012. TREDV were subcategorized based on additional information in the chief complaint data field. Tattoo-related emergency department infection visits (TREDV-I) were defined as TREDV containing the keywords (or word roots) of abscess, blisters, burn, cellulitis, crust, fever, growth, infection, irritation, itch, pimples, pus, or wound. The TREDV pain subcategory (TREDV-P) was defined by the keywords hurt and pain. The swelling subcategory (TREDV-S) was defined by edema, bump, knot, mass, swelling, or swollen. All remaining TREDV were categorized as other.

Descriptive statistics and the chi-square test were used to compare TREDV by subcategory, age, sex, and borough of residence. Rates of TREDV were calculated by age, sex, and borough using the appropriate subset of ED visits as the denominator, and compared using the chi-square test. Direct age adjustment using 2010 census population estimates was used to compare rates between NYC boroughs. Trends in the annual TREDV rates were determined using the chi-square test for trend. Data coding and descriptive analysis were performed with IBM SPSS Statistics (Rel. 17.0.0. 2008. Chicago: IBM). Epi Info version 3.5.3 (CDC, Atlanta) was used for TREDV rate comparison and trend analysis.

## Results

### Case finding

Thirty-one TREDV were identified from NYC syndromic surveillance data during November 1, 2012 through March 31, 2013. The median age of TREDV cases was 24 years (range 16 to 48 years) and 20 (65%) were female. The highest number of TREDV cases lived in Manhattan (10 [32%]), followed by the Bronx (7 [23%]), Queens (6 [19%]), Brooklyn (4 [13%]), Staten Island (2 [7%]), and other (2 [7%]). Interviews were completed for 14 (45%) cases. Of the 17 cases not interviewed, 11 had incorrect phone numbers and six did not respond after three attempts. Those interviewed did not differ significantly from those who could not be reached on age, sex, or borough of residence.

Thirteen (93%) interviewed patients did not meet the definition of a possible case of NTM infection. One TREDV patient with symptoms lasting 22 weeks was referred to a dermatologist and was subsequently diagnosed with methicillin resistant *Staphylococcus aureus* (MRSA).

Although NYC laboratories reported 13 isolates of NTM from skin or soft tissue specimens from January 1, 2012 through March 31, 2013, none of the NTM isolates were from patients with recent tattoos. No reports of unusual clusters of tattoo-related complications were reported to the health department from health care providers during this time.

### Five-year TREDV trends

A total of 577 TREDV were identified from 43 (88%) NYC EDs from 2008 to 2012 (Table 1). TREDV represented 0.003% of ED visits for adults ages 18 to 64 during the interval. The median age of all cases was 25 years (range 10 to 68 years) and 332 (58%) were female. Thirty-seven (6%) TREDV occurred in persons younger than 18 years of age.

**Table 1:** Descriptive characteristics of tattoo-related emergency department visits (TREDV), New York City, 2008–2012.

Study parameter	TREDV (n = 577)	TREDV-I* (n = 380)	TREDV All Other Categories (n = 197)
	n (%)	n(%)	n(%)
**Age**			
<18	37 (6.4)	16 (4.1)	21 (10.7)
18–24	247 (42.8)	164 (43.2)	83 (42.1)
25–34	191 (33.1)	126 (33.2)	65 (33.0)
35–44	76 (13.2)	55 (14.5)	21 (10.7)
45–64	24 (4.2)	18 (4.7)	6 (3.0)
>64	2 (0.3)	1 (0.3)	1 (0.5)
**Sex**			
Men	245 (42.5)	156 (41.1)	89 (45.2)
Women	332 (57.5)	224 (58.9)	108 (54.8)
**Borough of Residence**			
Bronx	188 (32.6)	116 (30.5)	72 (36.5)
Brooklyn	164 (28.4)	116 (30.5)	48 (24.4)
Manhattan	114 (19.8)	70 (18.4)	44 (22.3)
Queens	66 (11.4)	46 (12.1)	20 (10.2)
Staten Island	22 (3.8)	17 (4.5)	5 (2.5)
Other	23 (4.0)	15 (3.9)	8 (4.1)

* Tattoo-related emergency department visits where an infection was suspected or identified (TREDV-I)

TREDV-I accounted for 380 (66%) of all TREDV. The remaining visits were categorized as follows: swelling (12%), pain (11%), and other (11%). Those categorized as TREDV-I did not differ significantly from those who were classified in all other categories on age, sex, or borough of residence.

TREDV rates were highest among those aged 18 to 24 years, followed by those aged 25 to 34 years, 35 to 44 years, and 45 to 64 years. The 18 to 24 year age-group had a significantly greater rate of TREDV compared to all other age groups (Table 2). Among adults 18–64 years, TREDV rates were higher for women than men although the difference was not statistically significant (p = 0.06). Rates of TREDV for these adults were highest in the Bronx, followed by Manhattan, Brooklyn, Staten Island, and Queens (Table 2). The Bronx had a significantly greater rate of TREDV compared with Brooklyn, Staten Island, and Queens, but not Manhattan. Direct age-adjustment using the 2010 Census NYC population did not result in an appreciable difference in rates. One Bronx hospital saw 10% of all citywide TREDV (N = 58). There was no significant increase in TREDV rates from 2008 to 2012 (chi-square for trend = 0.13, p = 0.71) (Table 3).

**Table 2:** Rates of tattoo-related emergency department visits (TREDV) per total adult emergency department (ED) visits by age, sex, and borough of residence, New York City, 2008–2012.

Study parameter	TREDV	Total ED Visits (million)	Rate per 100,000 ED visits	p-value
**Age Group**				
18–24	247	2.36	10.4	ref
25–34	191	3.19	6.0	<0.001
35–44	76	2.56	3.0	<0.001
45–64	24	4.53	0.5	<0.001
**Sex**				
Men	221	5.69	3.9	ref
Women	317	6.94	4.6	0.062
**Borough of Residence**				
Bronx	171	3.07	5.6	ref
Brooklyn	158	3.62	4.4	0.026
Manhattan	103	2.27	4.5	0.095
Queens	62	2.27	2.7	<0.001
Staten Island	22	0.63	3.5	0.040

**Table 3:** Rates of tattoo-related emergency department visits (TREDV) per total adult emergency department (ED) visits, New York City, 2008–2012.

Year	TREDV	Total ED visits (Ages 18–64) (million)	TREDV rate per 100,000 ED visits
**2008**	96	2.36	4.1
**2009**	108	2.51	4.3
**2010**	111	2.53	4.4
**2011**	106	2.59	4.1
**2012**	117	2.64	4.4

## Discussion

We used syndromic surveillance to investigate the possibility of an outbreak of tattoo-associated NTM skin and soft tissue infections and describe tattoo-related ED visits in NYC. We determined that despite the recent report of two isolated cases of NTM in tattoo recipients, there was no evidence that an outbreak was occurring.

Since NTM is not reportable in NYC, options for this type of disease investigation are limited. The New York City health alert network (HAN), a system used to communicate messages to the medical community, has traditionally been used to help case finding of non-reportable diseases. A HAN alert was not used in this investigation because two cases occurring ten months apart did not warrant provider notification. However, providers are accustomed to reporting unusual manifestations of disease in which links to a procedure or commercial product are noted. Data from the Statewide Planning and Research Cooperative System for ED visits (ED SPARCS) has limited utility for outbreak investigations because of the lag time between event and the availability of data, the inability to perform keyword searches, and the absence of specific ICD-9/10 codes for tattoo-related infections. One limitation of our study is that we were only able to interview 45% of TREDV patients during the case finding portion of the investigation, so it remains possible that we missed possible tattoo-related NTM infections.

Tattoo-related complications have been described; information on the incidence of tattoo-related complications, however, is limited [3,6]. Rates of TREDV among adults were highest among Bronx residents and those ages 18 to 24. The higher TREDV rate among the 18 to 24 year age is consistent with findings from other studies [1,4]. There are several potential reasons why the TREDV rate is highest in Bronx residents, such as greater ED utilization for primary care or greater descriptiveness in syndromic data.

Most TREDV were subcategorized as infection-related, but the sensitivity and specificity of ED chief complaint data to identify skin and soft tissue infection is unknown. Complaints including “pus,” “cellulitis,” “fever,” “irritation,” “pimples,” and “itch” were used as a proxy for a diagnosis of “infection;” however the latter three may have been symptoms of allergic reaction, rather than infection. This could have resulted in an overestimation of infection-related TREDV. Likewise the inclusion of “hurt,” “pain,” “edema,” “bump,” “knot,” “mass,” “swelling,” and “swollen” in other categories may have missed tattoo-related infections. As all TREDV were included during the case finding phase this classification should not have affected our ability to detect the existence of an outbreak of NTM infections. This investigation only captured tattoo-related complications among those who sought medical care in an ED, which may be an underrepresentation of tattoo-related complications in NYC. However, had a large outbreak of NTM occurred we would likely have detected it through a combination of surveillance systems (ED syndromic, laboratory inquiry, provider reporting).

ED syndromic surveillance has additional limitations. Chief complaints may not include the word tattoo or may be inaccurate and/or incomplete. Word errors in chief complaint recording in the ED has been reported as high and can affect the retrieval performance of free-text syndromic surveillance data [7]. Chief complaints with common misspellings (e.g. “tatoo”) were included in this analysis, but those with typographical errors or uncommon abbreviations may not have been detected. Chief complaint coding, completeness and accuracy varied by hospital. For example, TREDV were identified in 88% of NYC EDs from 2008 to 2012. It is unknown whether the remaining 12% of hospitals had uncoded TREDV during this time. Similarly, the fact that 10% of all NYC TREDV occurred in one Bronx hospital may be in part the result of different coding practices or greater data completeness at this hospital as opposed to an actual increase proportion of TREDV. We did not survey dermatopathology laboratories that provide diagnostic services to outpatient dermatologists who may encounter patients seeking evaluation of infected tattoos. It is not known what proportion of patients might be evaluated by these providers, however, future investigations will incorporate specialty laboratories into surveillance.

We identified a single TREDV possibly due to NTM using ED syndromic surveillance data. Upon further investigation, the patient was not found to have NTM and we determined that no tattoo-associated NTM outbreak was occurring. In response to concerns about NTM infections among tattoo recipients, DOHMH sent a letter to all licensed tattoo artists advising them that when using water to dilute tattoo inks, sterile water must be used. Prospective surveillance from September 15, 2013 to December 31, 2013 did not uncover any additional suspect cases of tattoo-related NTM and has provided some reassurance that departmental recommendations not to dilute tattoo ink are being followed. An unintended consequence of our investigation was the identification of minors who received tattoos. As part of ongoing surveillance of ED tattoo visits, those identified in underaged individuals will be investigated for referral to the DOHMH licensing bureau.

Our investigation demonstrates that syndromic surveillance is a viable option for performing case finding when the event under surveillance is described by a unique word or phrase, but is not reportable by law and is otherwise difficult to capture. Additional data are needed to explore why TREDV rates are highest in the Bronx.
